# Self-Esteem Moderates the Effect of Compromising Thinking on Forgiveness Among Chinese Early Adolescents

**DOI:** 10.3389/fpsyg.2020.00104

**Published:** 2020-02-04

**Authors:** Wei Hong, Ru-De Liu, Yi Ding, Tian Po Oei, Xinchen Fu, Ronghuan Jiang, Shuyang Jiang

**Affiliations:** ^1^Beijing Key Laboratory of Applied Experimental Psychology, National Demonstration Center for Experimental Psychology Education (Beijing Normal University), Faculty of Psychology, Beijing Normal University, Beijing, China; ^2^Graduate School of Education, Fordham University, New York, NY, United States; ^3^School of Psychology, The University of Queensland, St Lucia, QLD, Australia

**Keywords:** compromising thinking, decisional forgiveness, emotional forgiveness, self-esteem, early adolescents

## Abstract

Forgiveness contributes to positive social relationships, which is critical for individual development, particularly for early adolescents. Most previous studies focused on the unique roles of cognitive factors (e.g., compromising thinking) and personality traits (e.g., self-esteem) in the process of developing forgiveness. However, sporadic research has examined their interactive effect on forgiveness from an integrated perspective. Given that forgiveness has been categorized into decisional and emotional forgiveness, this study aimed to examine the effects of compromising thinking on two types of forgiveness, and the moderating effects of self-esteem on the association between compromising thinking and forgiveness among early adolescents. A total of 1,009 Chinese primary and secondary school students (50.4% males; *M*_age_ = 11.75, *SD* = 1.27) were recruited to complete three self-reported questionnaires. The results showed that compromising thinking predicted decisional forgiveness but not emotional forgiveness. Furthermore, self-esteem was identified to moderate the conditional effects of compromising thinking on decisional and emotional forgiveness. These findings advance a better understanding of the construct and mechanism of forgiveness, which can provide insights for targeted forgiveness interventions among early adolescents, such as compromising thinking instructions and self-esteem enhancement programs.

## Introduction

Early adolescence is a developmental period in which social relationships become increasingly important and complex (Parker et al., 2006), and interpersonal problems become frequent and intense (Karimova, 2015; Ma et al., 2019). A critical element in assisting in maintaining, developing, and re-establishing interpersonal relationships is forgiveness, which facilitates individual prosocial development, especially for adolescents (McCullough, 2000; Karremans et al., 2011). Forgiveness refers to a prosocial change in emotions and behaviors after experiencing a transgression (McCullough, 2000). It is acknowledged as a principle within the ethical codes of various religious cultures, which has been substantiated to engender a variety of positive benefits, such as fewer health problems related to high blood pressure, elevated heart rate, and somatic symptoms (Karremans and Van Lange, 2008), lower levels of stress and depression (Lawler-Row and Piferi, 2006; Green et al., 2012), and higher levels of life satisfaction and psychological well-being (Worthington, 2005; Karremans and Van Lange, 2008). Thus, forgiveness is of great existential value and has gained increasing attention from researchers.

Forgiveness is known as a complex process that involves reducing negative motivation and increasing positive motivation after experiencing a transgression (Worthington, 2005). It can be categorized as *decisional forgiveness* and *emotional forgiveness*. The former refers to the behavioral intention to resist an unforgiving stance and to respond differently toward a transgressor; the latter refers to reduction of unforgiving-related negative emotions and replacement with positive prosocial emotions. These two processes have an essential distinction and interactively explain the process of forgiveness (Worthington et al., 2007b). Although numerous studies have recognized the importance of forgiveness, little research has focused on the subconstructs of forgiveness. Thus, this study focused on decisional and emotional forgiveness, instead of considering forgiveness as an integrated entity.

Most studies have focused almost exclusively on the unique roles of cognitive factors and individual traits in forgiveness (Zhang and Gu, 2009). For instance, adolescents with compromising thinking are more willing to forgive the experienced transgression (Lv et al., 2015). Also, adolescents with high self-esteem are more likely to grant forgiveness (Eaton et al., 2006; Yao et al., 2016). Recently, cognitive factors and personality traits have been proposed to interactively affect the process of forgiveness (Fehr et al., 2010). However, sporadic research provided empirical evidence, such that Miao et al. (2018) found that self-esteem moderated the effect of empathy on forgiveness among early adolescents. Importantly, empathy is associated with perspective-taking that may be related to dialectical thinking and compromise-focused thought (Lv et al., 2015); thus, the effects of compromising thinking on decisional and emotional forgiveness may be moderated by self-esteem. Taken together, this study aimed to examine the direct effect of compromising thinking and the moderating effect of self-esteem on decisional and emotional forgiveness among early adolescents.

### Compromising Thinking and Forgiveness

Cognitive factors have been identified as critical contributing factors in explaining forgiveness in the stress and coping model of forgiveness (Worthington, 2006), and it is possible that compromising thinking may be a potential predictor. Compromising thinking refers to a general inclination to having a middle ground attitude rather than extreme propositions when confronting contradictions, which is identified as an important dimension subordinated to holistic thinking (Choi et al., 2007). That is, people with compromise-focused thought tend to exhibit high tolerance of contradictions, have high preferences for naïve dialecticism and constructive strategies, and pursue compromising solutions to problems (Nisbett et al., 2001; Spencer-Rodgers et al., 2010). This thinking style may be applicable to the context in which a person encounters a transgression. Adolescents with compromise-focused thought are oriented to avoid interpersonal conflicts, hold non-extreme attitudes, and try to maintain social harmony (Fu et al., 2004). During this process, they may inhibit impulsive revenge behaviors, which seems to increase decisional forgiveness (Lv et al., 2015).

Furthermore, adolescents with compromise-focused thought tend to think through a holistic perspective; also, they may dialectically reconsider the experienced transgression. This process may contribute to thoughts that there may be other contributing factors involved (e.g., It may be not totally caused by the offender), and/or there may be reciprocity (e.g., I may be forgiven in case I unintentionally offend others) (Lv et al., 2015). In this sense, those adolescents may try to reduce negative affection and grant emotional forgiveness (Donovan and Priester, 2017; Smith et al., 2019). In support of this, empirical research has found that adolescents who score high in compromising thinking are more likely to forgive the offender after being offended compared with those who score low in compromising thinking (Lv et al., 2015). Additionally, indirect experimental evidence showed that priming compromising thinking could reduce aggressive tendencies, state anger, and hostility that are associated with unforgiveness (Zhang et al., 2011; Fatfouta et al., 2015). Based on this review of the literature, the following hypotheses guided this study:

**H1a:** Compromising thinking is positively associated with decisional forgiveness.

**H1b:** Compromising thinking is positively associated with emotional forgiveness.

### Self-Esteem as a Moderator

Despite the potential role compromising thinking plays in explaining the process of forgiveness, the extent of association may vary with individual differences, such as how an individual feels about and interprets a situation after experiencing a transgression (Lv et al., 2015). As stated earlier, self-esteem refers to the extent to which an individual identifies and values the self (Rosenberg, 1965). It has been regarded as a regulator of self-threat to influence the perceptions and interpretation of the self and the world (Trzesniewski et al., 2006; Sciangula and Morry, 2009). That is, high levels of self-esteem not only directly influences forgiveness, but also has a moderating effect during the process of forgiveness (Eaton et al., 2006; Yao et al., 2016; Miao et al., 2018).

Specific to transgression contexts, people with low self-esteem feel insecure and have low self-confidence and fragile self-worth; they also show high interpersonal sensitivity and possibly treat being offended as a threat to self-worth (Eaton et al., 2006). After experiencing a transgression, adolescents with low self-esteem may automatically activate a psychological defense system. Thus, they are more likely to react with extreme behaviors (e.g., revenge) and feel as though they are not being respected even though they have compromise-focused thought (Fehr et al., 2010). That is, under the condition of low levels of self-esteem, compromising thinking may weakly affect decisional and emotional forgiveness.

In contrast, people with high self-esteem have stable self-cognition and are less influenced by the external environment (Deci and Ryan, 1995); they are less likely to overreact and to use striking-back strategies to enhance the self. In this sense, their inner cognitive thinking may determine how to cope with the transgression. Those high self-esteem adolescents with compromise-focused thought tend to hold non-extreme attitudes and are more willing to grant decisional and emotional forgiveness (Lv et al., 2015; Yao et al., 2016). In support of the moderating role of self-esteem, empirical studies have found that social-cognitive factors (e.g., empathy) have a stronger effect on forgiveness among early adolescents with high self-esteem compared with those with low self-esteem (Miao et al., 2018). Also, forgiveness interventions (i.e., instructing how to interpret transgressions) have a stronger effect on positive emotions toward the offender among women with high self-esteem compared with those with low self-esteem (Cardi et al., 2007). On the basis of the above findings, we proposed the following hypotheses:

**H2a:** Self-esteem moderates the effect of compromising thinking on decisional forgiveness. That is, compromising thinking has a stronger association with decisional forgiveness with high self-esteem compared with those with low self-esteem.

**H2b:** Self-esteem moderates the effect of compromising thinking on emotional forgiveness. That is, compromising thinking has a stronger association with emotional forgiveness with those who have high self-esteem compared with those with low self-esteem.

### The Present Study

By reviewing the existing literature, cognitive factors and personality traits have been argued to interactively influence the process of forgiveness (Zhang and Gu, 2009; Fehr et al., 2010). Compromising thinking as an inclination to middle ground attitude may contribute to promoting decisional and emotional forgiveness after experiencing a transgression. Similarly, self-esteem as a regulator of self-threat influences the perceptions of transgressions, which may moderate the associations between compromising thinking and the two types of forgiveness. Considering that compromising thinking is a manifestation of Chinese culture characteristics, in which Chinese people have more compromise-focused thought compared with Westerners (as they are deeply influenced by the Confucian philosophy) (Fu et al., 2004; Spencer-Rodgers et al., 2010), this study focused on Chinese contexts and could contribute to a better understanding of forgiveness from a cultural perspective. Additionally, it is known that age is positively associated with forgiveness due to moral reasoning development and psychological maturity (Toussaint et al., 2001; Fehr et al., 2010). However, most of the previous studies were limited to adolescence and adult participants; the findings might be of somewhat limited value. To this end, the present study aimed to examine the direct effect of compromising thinking and the moderating effect of self-esteem on decisional and emotional forgiveness among early adolescents.

## Materials and Methods

### Participants and Procedures

Participants were 1,009 students (509 males = 50.4%; 28 students did not report gender information) who were recruited from two ordinary primary and secondary schools in Beijing, China, with an age range from 10 to 15 years (*M* = 11.75, *SD* = 1.27). Participants consisted of 24.7% fourth graders, 33.0% fifth graders, 24.2% sixth graders, 13.1% seventh graders, and 5.0% eighth graders.

This research was approved by the Academic Ethics Committee of the Faculty of Psychology at Beijing Normal University and was implemented by research assistants in the fall semester of 2018. Prior to the formal investigation, six research assistants interviewed a total of 30 students randomly selected from primary and secondary schools (not including data collection participants). This process helped to clarify the words, statements, and instructions that were fit for students, especially for those in lower grades, which could avoid possible result bias (Fowler, 2002). Students voluntarily participated in this study. Written informed consent was obtained from participants’ parents and legal guardians before the study. The informed consent and assent forms informed the participants that this investigation was anonymous and confidential, and the data would be used only for academic research. Compromising thinking, self-esteem, and decisional and emotional forgiveness were measured in sequence by the same pencil-and-paper questionnaires in the regular classrooms; the time estimated to complete the self-reported questionnaires was approximately 15 min.

### Measures

#### Compromising Thinking

Compromising thinking style was assessed by a subscale of the Analysis-Holism Thinking Scale (Choi et al., 2007) and reflected a general middle ground attitude toward contradictions. The scale had five items with a single dimension (e.g., “It is more desirable to take the middle ground than go to extremes”). Participants rated to what extent they agreed with each item on a 7-point Likert scale, ranging from 1 (*strongly disagree*) to 7 (*strongly agree*). Higher scores indicated higher tendencies to engage in compromising thinking. The results of confirmatory factor analysis (CFA) supported the one-dimension construct validity, chi-square values, χ^2^ (4) = 47.75, the comparative fit index (CFI) = 0.97, the Tucker-Lewis fit index (TLI) = 0.93, the root mean square error of approximation (RMSEA) = 0.10, and the standardized root-mean-square residual (SRMR) = 0.03. Validity evidence showed moderate correlations with relevant measures such as the Global Style Scale and the Rahim Organizational Conflict Inventory–II (Choi et al., 2007). Additionally, the scale in this study had good internal consistency (Cronbach’s α = 0.81).

#### Self-Esteem

The level of self-esteem was measured by a validated Chinese version of the Rosenberg Self-Esteem Scale (Yang and Wang, 2007). It had 10 items (e.g., “On the whole, I am satisfied with myself”) and used a 5-point Likert scale (1 = *completely disagree*, 5 = *completely agree*), with higher scores indicating higher levels of self-esteem. The results of CFA showed that the validity of this scale was acceptable, χ^2^ (28) = 114.88, CFI = 0.99, TLI = 0.98, RMSEA = 0.06, SRMR = 0.03. The scale was moderately correlated with the scale of assessing self-consistency and congruency (Yang and Wang, 2007). Additionally, the present sample revealed good internal consistency of the Rosenberg Self-Esteem Scale (Cronbach’s α = 0.88).

#### Forgiveness

Two distinguishable forgiveness processes are decisional forgiveness and emotional forgiveness, which were assessed by the Decisional Forgiveness Scale and the Emotional Forgiveness Scale, respectively (Worthington et al., 2007a; Dorn et al., 2013), which has been validated in the Chinese context (Chi et al., 2011). The former scale contains eight items with a two-component structure, prosocial intention (e.g., “If I see him or her, I will act friendly”), and inhibition of harmful intention (e.g., “I will not seek revenge upon him/her”). The latter scale also has eight items with a two-component structure, reduction of negative emotion (e.g., “I no longer feel upset when I think of him or her”), and presence of positive emotion (e.g., “I feel sympathy toward him/her”). After participants were instructed “Think about someone who has hurt or offended you, and recall the details of the transgression,” they responded to each item on a 5-point Likert scale (1 = *completely disagree*, 5 = *completely agree*), with higher scores indicating higher tendencies to engage in decisional and emotional forgiveness. The results of CFA supported the two-dimension construct validity of the Decisional Forgiveness Scale, χ^2^ (17) = 137.48, CFI = 0.96, TLI = 0.93, RMSEA = 0.08, SRMR = 0.05, and the Emotional Forgiveness Scale, χ^2^ (16) = 119.17, CFI = 0.96, TLI = 0.93, RMSEA = 0.08, SRMR = 0.06. The Decisional and Emotional Forgiveness Scales were both strongly correlated with other measures of interpersonal forgiveness, trait, and state forgiveness (Hook, 2007). The two scales had good construct validity and acceptable internal consistency (Cronbach’s α = 0.77, 0.68, respectively).

### Data Analysis

Descriptive analyses and correlations were conducted using SPSS 19.0. The direct effect of compromising thinking and the moderating role of self-esteem on forgiveness were examined using structural equation modeling (SEM) in Mplus 7.1. Moreover, the missing data were handled by maximum likelihood (ML) estimates in the analyses. The model fit was evaluated by χ^2^, CFI, TLI, RMSEA, and SRMR. Notably, CFI and TLI values were larger than 0.9, and RMSEA and SRMR values were less than 0.08, indicating an acceptable model fit (Wen et al., 2004).

## Results

### Descriptive Statistics

Means, standard deviations, and Pearson correlations for all variables are presented in Table 1. Age was not correlated with compromising thinking and self-esteem, but was negatively correlated with both decisional and emotional forgiveness, suggesting that it should be considered as a covariate. Compromising thinking, self-esteem, and decisional and emotional forgiveness were positively correlated with each other, except for the non-significant correlations between compromising thinking and emotional forgiveness, and between self-esteem and emotional forgiveness.

**TABLE 1:** Means, Standard Deviations and Correlations.

	***M***	***SD***	**1**	**2**	**3**	**4**	**5**	**6**	**7**	**8**	**9**
1 Age	11.75	1.27	–								
2 Compromising	26.01	6.79	0.03	–							
3 Self-esteem	36.80	8.55	–0.02	0.31***	–						
4 Decisional	28.03	6.69	−0.08*	0.16***	0.15***	–					
5 Prosocial	13.52	3.37	−0.08*	0.16***	0.10**	0.85***	–				
6 Inhibition	14.51	4.19	−0.07*	0.13***	0.17***	0.91***	0.55***	–			
7 Emotional	21.80	5.97	−0.13***	0.01	0.01	0.49***	0.44***	0.42***	–		
8 Positive	9.89	4.23	−0.09**	0.04	–0.04	0.31***	0.34***	0.23***	0.84***	–	
9 Negative	11.91	3.37	−0.12***	–0.04	0.08*	0.46***	0.34***	0.46***	0.72***	0.22***	–

Compromising = Compromising thinking, Decisional = Decisional forgiveness, Prosocial = Prosocial intention, Inhibition = Inhibition of harmful intention, Emotional = Emotional forgiveness, Positive = Presence of positive emotion, Negative = Reduction of negative emotion. Prosocial intention and inhibition of harmful intention as the subconstructs of decisional forgiveness; presence of positive emotion and reduction of negative emotion as the subconstructs of emotional forgiveness *p < 0.05, **p < 0.01, ***p < 0.001.

### The Direct Effect of Compromising Thinking

Compromising thinking and self-esteem can be handled as manifest variables because they have a one-dimensional and homogeneous construct. Decisional and emotional forgiveness have multiple dimensions, they thus can be parceled as latent variables according to subordinated dimensions in SEM analyses (Wu and Wen, 2011). To test hypotheses H1a and H1b, a direct model was conducted and showed a satisfactory model fit, χ^2^ (4) = 11.00, CFI = 0.99, TLI = 0.97, RMSEA = 0.04, SRMR = 0.02. The model indicated that compromising thinking positively predicted decisional forgiveness (β = 0.18, *p* < 0.001) but failed to predict emotional forgiveness (*p* = 0.89 > 0.05) after controlling for the effect of age. The finding indicated compromising thinking was associated with decisional forgiveness but not with emotional forgiveness, supporting H1a, but not supporting H1b.

### The Moderating Effect of Self-Esteem

**FIGURE 1:**
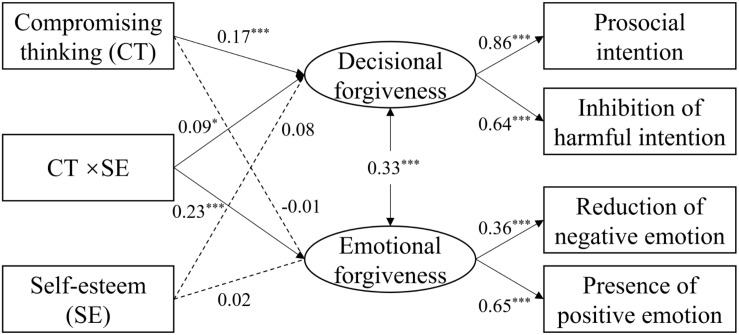
A moderating model of self-esteem in the associations between compromising thinking and forgiveness. The effect of age was controlled. All coefficient estimates are completely standardized. *p < 0.05, ***p < 0.001.

In regard to the hypotheses of self-esteem (H2a, H2b), the moderating model had a good model fit, χ^2^ (8) = 23.93, CFI = 0.98, TLI = 0.94, RMSEA = 0.05, SRMR = 0.02. As shown in Figure 1, the results suggested that the interaction term (i.e., a product of compromising thinking and self-esteem) predicted both decisional and emotional forgiveness after controlling for the effect of age. In addition, bias-corrected bootstrap tests with 1000 deriving samples were adopted. Neither the confidence interval (CI) of the interaction term on decisional (90% CI [0.001, 0.15]) and emotional forgiveness (95% CI [0.03, 0.13]) included zero, indicating the significant moderating effects of self-esteem.

**FIGURE 2:**
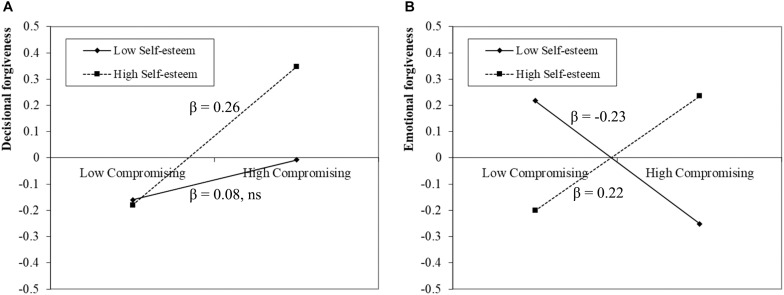
Self-esteem moderated the effects of compromising thinking on decisional and emotional forgiveness. **(A)** indicates the interactive effect on decisional forgiveness. **(B)** indicates the interactive effect on emotional forgiveness. ns, non-significant.

To further clarify the essence of the interaction effect, a simple slope analysis was conducted. Specifically, participants were divided into two counterparts (i.e., High = *M* + *SD*; Low = *M*−*SD*) on the basis of the levels of the moderator. As shown in Figure 2A, among early adolescents with low levels of self-esteem, compromising thinking did not predict decisional forgiveness (β = 0.08, 95% CI [−0.04, 0.17]), while the prediction became significant among early adolescents with high levels of self-esteem (β = 0.26, 95% CI [0.11, 0.35]). Importantly, further examination of the slopes under different levels of self-esteem demonstrated marginal significance (90% CI [0.02, 0.30]), which suggested that self-esteem moderated the effect of compromising thinking on decisional forgiveness, supporting H2a.

Similarly, as shown in Figure 2B, among early adolescents with low levels of self-esteem, compromising thinking negatively predicted emotional forgiveness (β = −0.23, 95% CI [−0.14, −0.03]). In contrast, among early adolescents with high levels of self-esteem, compromising thinking positively predicted emotional forgiveness (β = 0.22, 95% CI [0.01, 0.15]). Additional examination of slopes demonstrated statistical significance (95% CI [0.07, 0.26]), which indicated self-esteem moderated the effect of compromising thinking on emotional forgiveness, supporting H2b.

## Discussion

This study was one of a few studies that simultaneously examined the effects of cognitive factors (e.g., compromising thinking) and personality traits (e.g., self-esteem) on decisional and emotional forgiveness among primary and secondary school students. Based on a large sample of early adolescents, the results revealed slightly negative correlations between age and two types of forgiveness. Furthermore, compromising thinking positively predicted decisional forgiveness, but it did not directly predict emotional forgiveness. More importantly, the conditional predictions were moderated by individual difference in self-esteem. In sum, these findings contribute to further understanding of the construct and mechanism of forgiveness within Chinese contexts, which can lend credence to prosocial development interventions among young people.

### The Relation Between Age and Forgiveness

This study showed weak and negative correlations between age and decisional forgiveness/emotional forgiveness among primary and secondary school students. The negative relation was consistent with the findings among fourth, seventh, and ninth grader samples (Goss, 2007). Although the previous study found that age was positively associated with forgiveness (Toussaint et al., 2001), the discrepancy may be due to the age span of the sample. That is, Toussaint’s study investigated participants aged from 18 to over 65 years, whereas this study focused on early adolescence aged 10–15 years. As earlier, turning from late childhood to early adolescence, interpersonal conflicts become more frequent and intense (Karimova, 2015; Ma et al., 2019). Thus, early adolescents seem to show a slight decline in decisional and emotional forgiveness, although they may be becoming psychologically mature. Taken together, these findings partially supported the notion that prosocial behaviors show an initial decline during early adolescence and a subsequent increase during adulthood (Luengo Kanacri et al., 2013). For another thing, the weak correlations coincided with the meta-analysis results in which relationship between age and forgiveness (*r* = 0.06) was so small that it can be nearly negligible (Fehr et al., 2010). Future studies (e.g., expanding age span, using longitudinal research) are warranted in order to further reveal the association between age and forgiveness.

### Compromising Thinking, Self-Esteem, and Decisional Forgiveness

The results showed that compromising thinking positively predicted decisional forgiveness after the effect of age was controlled, consistent with the previous research (Lv et al., 2015). Influenced by the doctrine of the mean of the Confucian philosophy, such as the proverb “a bad compromise is better than a good lawsuit,” Chinese people are prone to adopt a middle ground attitude and find compromising solutions when they are confronted with contradictions (Nisbett et al., 2001). Specific to a transgression context, when being offended by someone, Chinese early adolescents with compromise-focused thought may be less likely to react with extreme behaviors as they are oriented against interpersonal conflict and toward social harmony (Fu et al., 2004). Thus, they may not seek revenge upon the offender, and even try to treat him/her as usual to maintain harmonious relationships (Lv et al., 2015). It seemed possible that compromising thinking was positively associated with decisional forgiveness among early adolescents.

Furthermore, self-esteem moderated the effect of compromising thinking on decisional forgiveness. That is, compromising thinking did not predict decisional forgiveness among early adolescents with low self-esteem, but it positively predicted decisional forgiveness among early adolescents with high self-esteem. Consistent with the previous research, the role of social-cognitive factors in predicting forgiveness strengthened with increased self-esteem (Miao et al., 2018). Early adolescents with low self-esteem feel insecure and have low self-worth; they may give priority to protecting their threatened self-esteem (Eaton et al., 2006). Those with compromise-focused thought seem not to resist seeking revenge and not to express a forgiving attitude to the transgressor, because they tend to self-protect even though they have compromise-focused thought toward the transgression. In contrast, early adolescents with high self-esteem have relatively secure and stable self-cognition; they may attenuate negative value of the offense and not treat it as a threat to the self (Eaton et al., 2006). Their compromise-focused thought may encourage them to hold a middle ground attitude and to find harmonious resolutions after experiencing a transgression (Lv et al., 2015). In short, it was plausible that compromising thinking was not associated with decisional forgiveness among early adolescents with low self-esteem, but it was positively associated with decisional forgiveness among early adolescents with high self-esteem.

### Compromising Thinking, Self-Esteem, and Emotional Forgiveness

Although compromising thinking did not directly predict emotional forgiveness after the effect of age was controlled, self-esteem moderated the association between compromising thinking and emotional forgiveness. That is, compromising thinking negatively predicted decisional forgiveness among early adolescents with low self-esteem, whereas it positively predicted decisional forgiveness among early adolescents with high self-esteem. As stated earlier, self-esteem to some extent influences the perceptions of external and subsequent behaviors (Trzesniewski et al., 2006; Sciangula and Morry, 2009). In turn, these perceptions and thoughts may regulate how to interpret and appraise transgressions, as described in the stress and coping model of forgiveness (Strelan and Covic, 2006; Worthington, 2006). For instance, after suffering from being offended, early adolescents with low self-esteem may experience emotional shifts and exhibit high hostility (Zeigler-Hill et al., 2011). Those people may appraise the offense as an act of provocation and regard emotional inaction as a manifestation of weakness (Fehr et al., 2010). Afterward, they may feel angry and resentful toward the transgression (e.g., emotional unforgiveness), although they have compromise-focused thought.

In contrast, early adolescents with high self-esteem have sufficient psychological resources; they may appraise the incident as a challenge for self-enhancement and believe in prosocial changes as a way of spirituality-shaping and personal growth (Lawler-Row and Piferi, 2006; Strelan and Covic, 2006). In this sense, those people with compromise-focused thought are more likely to exhibit positive emotions and be more willing to grant emotional forgiveness. By and large, it was plausible that compromising thinking was negatively associated with emotional forgiveness among early adolescents with low self-esteem, but it was positively associated with emotional forgiveness among early adolescents with high self-esteem.

### Limitations, Implications and Future Directions

Several limitations of this study should be noted. First, a cross-sectional design cannot make causal inferences; thus, future researchers might use an experimental design to further examine its effect on forgiveness. For example, compromising thinking can be primed by relevant materials (Zhang et al., 2011), and forgiveness can be measured by the Forgiveness Implicit Association Test (Goldring and Strelan, 2017). Second, compromising thinking, to a large extent, is a characteristic of East Asians relative to Westerners (Nisbett et al., 2001); thus, future research could focus on cultural differences of thinking styles and its effects on forgiveness after transgressions. Third, although the Decisional and Emotional Scales have adequate reliability and validity, there is a possibility that participants may consider different types of transgressions, which might potentially influence the final results. Thus, the measures should be further improved and enhanced in any future research. Fourth, age appears to be positively related to forgiveness due to psychological maturity (Toussaint et al., 2001), but it was found to have weak and negative correlations in this study. Future studies could investigate people in a larger age span, which might contribute to validating the correlation and advancing a better understanding of the developmental process of forgiveness. Finally, given that forgiveness involves a transgressor, a victim, and sometimes observers and other social elements (Worthington, 2005), forgiveness processes may vary with situational characteristics (Fehr et al., 2010). Future researchers could consider other moderating mechanisms to enhance and integrate the models of forgiveness.

Despite these limitations, several theoretical and practical implications of this study should be noted. This study found a slightly negative relationship between age and forgiveness, which complements the previous research that mainly focused on adolescence and adulthood. Moreover, this study was, to date, the first piece of research to examine the effects of compromising thinking on decisional and emotional forgiveness, and the moderating effects of self-esteem on the association. On the one hand, these findings supported the unique and distinct constructs of forgiveness (Worthington et al., 2007b). On the other hand, this study provided empirical evidence to substantiate that cognitive factors and personal traits interact on the process of forgiveness (Zhang and Gu, 2009; Fehr et al., 2010).

In regards to targeted forgiveness interventions for early adolescents, compromising thinking can be improved by naïve dialecticism training, which may be instrumental in reconciling non-constructive cognitions and alleviate non-adaptive emotions, thereby further avoiding social conflicts, fostering forgiveness, reducing somatic symptoms, and increasing psychological well-being (Karremans and Van Lange, 2008). To be specific, parents, teachers, and mental health workers can guide students to think in a dialectical way, instruct them to hold a holistic perspective, and educate them to seek constructive solutions (Zhang et al., 2011). These processes may be conducive to reconciliating negative experiences (e.g., betrayal traumas) and facilitate adaptive coping strategies (Boyraz et al., 2019). For another thing, self-esteem was found not only as a contributing factor of forgiveness, but also as a potential moderator to increase the effects of cognitive factors on forgiveness. Families and schools could implement self-esteem enhancement programs, which are beneficial to ameliorate how students perceive and react after experiencing a transgression, and then promote the occurrence of decisional and emotional forgiveness, especially true among early adolescents with compromise-focused thought.

## Data Availability Statement

The datasets generated for this study are available on request to the corresponding author.

## Ethics Statement

The studies involving human participants were reviewed and approved by the Academic Ethics Committee of the Faculty of Psychology at Beijing Normal University. Written informed consent to participate in this study was provided by the participants’ legal guardian/next of kin.

## Author Contributions

WH and R-DL designed the study, analyzed the data, and drafted the manuscript. WH, YD, and TO revised the manuscript. XF, RJ, and SJ collected the data. All co-authors participated in the discussion of the results.

## Conflict of Interest

The authors declare that the research was conducted in the absence of any commercial or financial relationships that could be construed as a potential conflict of interest.

## References

[B1] Boyraz G. Ferguson A. N. Zaken M. D. Baptiste B. L. Kassin C. (2019). Do dialectical self-beliefs moderate the indirect effect of betrayal traumas on posttraumatic stress through self-compassion? *Child Abuse Negl.* 96:104075. 10.1016/j.chiabu.2019.104075 31336237

[B2] Cardi M. Milich R. Harris M. J. Kearns E. (2007). Self-esteem moderates the response to forgiveness instructions among women with a history of victimization. *J. Res. Pers.* 41 804–819. 10.1016/j.jrp.2006.09.007

[B3] Chi P.-L. Du H.-F. Lam D. O. (2011). Mental health following partner’s extramarital involvement: role of decisional forgiveness and emotional forgiveness [in Chinese]. *Chin. J. Clin. Psychol.* 19 331–334. 10.16128/j.cnki.1005-3611.2011.03.019

[B4] Choi I. Koo M. Jong An C. (2007). Individual differences in analytic versus holistic thinking. *Pers. Soc. Psychol. Bull.* 33 691–705. 10.1177/0146167206298568 17440200

[B5] Deci E. L. Ryan R. M. (1995). “Human autonomy: the basis for true self-esteem,” in *Efficacy, Agency, and Self-Esteem*, ed. Kernis M. H., (New York, NY: Plenum).

[B6] Donovan L. A. N. Priester J. R. (2017). Exploring the psychological processes underlying interpersonal forgiveness: the superiority of motivated reasoning over empathy. *J. Exp. Soc. Psychol.* 71 16–30. 10.1016/j.jesp.2017.02.005

[B7] Dorn K. Hook J. N. Davis D. E. Van Tongeren D. R. Worthington E. L. (2013). Behavioral methods of assessing forgiveness. *J. Posit. Psychol.* 9 75–80. 10.1080/17439760.2013.844267

[B8] Eaton J. Ward Struthers C. Santelli A. G. (2006). Dispositional and state forgiveness: the role of self-esteem, need for structure, and narcissism. *Pers. Individ. Differ.* 41 371–380. 10.1016/j.paid.2006.02.005

[B9] Fatfouta R. Gerlach T. M. Schröder-Abé M. Merkl A. (2015). Narcissism and lack of interpersonal forgiveness: the mediating role of state anger, state rumination, and state empathy. *Pers. Individ. Differ.* 75 36–40. 10.1016/j.paid.2014.10.051

[B10] Fehr R. Gelfand M. J. Nag M. (2010). The road to forgiveness: a meta-analytic synthesis of its situational and dispositional correlates. *Psychol. Bull.* 136 894–914. 10.1037/a0019993.supp 20804242

[B11] Fowler F. J. J. (2002). *Survey Research Methods*, 3rd Edn Thousand Oaks, CA: Sage Publications.

[B12] Fu H. Watkins D. Hui E. K. P. (2004). Personality correlates of the disposition towards interpersonal forgiveness: a Chinese perspective. *Int. J. Psychol.* 39 305–316. 10.1080/00207590344000402

[B13] Goldring J. Strelan P. (2017). The forgiveness implicit association test. *Pers. Individ. Differ.* 108 69–78. 10.1016/j.paid.2016.12.006

[B14] Goss S. M. (2007). *The Influence of Friendship Quality and Commitment on the Empathy-Forgiveness Relationship in Children and Adolescents.* Doctoral dissertation, University of Nebraska, Lincoln, NE.

[B15] Green M. Decourville N. Sadava S. (2012). Positive affect, negative affect, stress, and social support as mediators of the forgiveness-health relationship. *J. Soc. Psychol.* 152 288–307. 10.1080/00224545.2011.603767 22558825

[B16] Hook J. N. (2007). *Forgiveness, Individualism, and Collectivism.* Doctoral dissertation, Virginia Commonwealth University, Richmond, VA.

[B17] Karimova L. S. (2015). Prevention of interpersonal conflicts in teenagers’ environment. *Procedia Soc. Behav. Sci.* 191 1843–1847. 10.1016/j.sbspro.2015.04.693

[B18] Karremans J. C. Regalia C. Paleari F. G. Fincham F. D. Cui M. Takada N. (2011). Maintaining harmony across the globe: the cross-cultural association between closeness and interpersonal forgiveness. *Soc. Psychol. Pers. Sci.* 2 443–451. 10.1177/1948550610396957

[B19] Karremans J. C. Van Lange P. A. M. (2008). Forgiveness in personal relationships: its malleability and powerful consequences. *Eur. Rev. Soc. Psychol.* 19 202–241. 10.1080/10463280802402609

[B20] Lawler-Row K. A. Piferi R. L. (2006). The forgiving personality: describing a life well lived? *Pers. Individ. Differ.* 41 1009–1020. 10.1016/j.paid.2006.04.007

[B21] Luengo Kanacri B. P. Pastorelli C. Eisenberg N. Zuffiano A. Caprara G. V. (2013). The development of prosociality from adolescence to early adulthood: the role of effortful control. *J. Pers.* 81 302–312. 10.1111/jopy.12001 22924862

[B22] Lv M. Pan J. Zheng Q. Xue H. Feng Z. (2015). The impact of compromising thinking on forgiveness and self-forgiveness in college students [in Chinese]. *China J. Health Psychol.* 23 71–74. 10.13342/j.cnki.cjhp.2015.01.020

[B23] Ma T. L. Zarrett N. Simpkins S. Vandell D. L. Jiang S. (2019). Brief report: patterns of prosocial behaviors in middle childhood predicting peer relations during early adolescence. *J. Adolesc.* 78 1–8. 10.1016/j.adolescence.2019.11.004 31790833

[B24] McCullough M. E. (2000). Forgiveness as human strength: theory, measurement, and links to well-being. *J. Soc. Clin. Psychol.* 19 43–55. 10.1521/jscp.2000.19.1.43

[B25] Miao L. Zhao K. Yang M. Lei X. Liu S. Zhang L. (2018). Parent-child attachment and interpersonal forgiveness among junior high school students: a moderated mediation model [in Chinese]. *Psychol. Dev. Educ.* 34 264–272. 10.16187/j.cnki.issn1001-4918.2018.03.02

[B26] Nisbett R. E. Peng K. Choi I. Norenzayan A. (2001). Culture and systems of thought: holistic versus analytic cognition. *Psychol. Rev.* 108 291–310. 10.1037/0033-295X.108.2.291 11381831

[B27] Parker J. G. Rubin K. H. Erath S. A. Wojslawowicz J. C. Buskirk A. A. (2006). “Peer relationships, child development, and adjustment: a developmental psychopathology perspective,” in *Developmental Psychopathology Theory and Methods*, 2nd Edn, eds Cicchetti D. Cohen D. J., (New York, NY: Wiley), 96–161.

[B28] Rosenberg M. (1965). *Society and the Adolescent Self-Image.* Princeton, NJ: Princeton University Press.

[B29] Sciangula A. Morry M. M. (2009). Self-esteem and perceived regard: how I see myself affects my relationship satisfaction. *J. Soc. Psychol.* 149 143–158. 10.3200/SOCP.149.2.143-158 19425354

[B30] Smith A. McCauley T. G. Yagi A. Yamaura K. Shimizu H. McCullough M. E. (2019). Perceived goal instrumentality is associated with forgiveness: a test of the valuable relationships hypothesis. *Evol. Hum. Behav.* 41 58–68. 10.1016/j.evolhumbehav.2019.09.003

[B31] Spencer-Rodgers J. Williams M. J. Kaiping P. (2010). Cultural differences in expectations of change and tolerance for contradiction: a decade of empirical research. *Pers. Soc. Psychol. Rev.* 14 296–312. 10.1177/1088868310362982 20435801

[B32] Strelan P. Covic T. (2006). A review of forgiveness process models and a coping framework to guide future research. *J. Soc. Clin. Psychol.* 25 1059–1085. 10.1521/jscp.2006.25.10.1059

[B33] Toussaint L. L. Williams D. R. Musick M. A. Everson S. A. (2001). Forgiveness and health: age differences in a US probability sample. *J. Adult Dev.* 8 249–257. 10.1023/A:1011394629736

[B34] Trzesniewski K. H. Donnellan M. B. Moffitt T. E. Robins R. W. Poulton R. Caspi A. (2006). Low self-esteem during adolescence predicts poor health, criminal behavior, and limited economic prospects during adulthood. *Dev. Psychol.* 42 381–390. 10.1037/0012-1649.42.2.381 16569175

[B35] Wen Z. L. Hau K. T. Marsh H. W. (2004). Structural equation model testing: cutoff criteria for goodness of fit indices and chi-square test [in Chinese]. *Acta Psychol. Sin.* 36 186–194. 10.1007/BF02911031

[B36] Worthington E. L. Jr. (2005). *Handbook of Forgiveness.* New York, NY: Brunner-Routledge.

[B37] Worthington E. L. Jr. (2006). “A stress-and-coping theory of forgiveness and relevant evidence,” in *Forgiveness and Reconciliation: Theory and Application*, ed. Worthington E. L. J., (Oxon: Routledge Press), 15–108.

[B38] Worthington E. L. Jr. Hook J. N. Utsey S. O. Williams J. K. Neil R. L. (2007a). “Decisional and emotional forgiveness,” in *Paper Presented at the International Positive Psychology Summit*, Washington, DC.

[B39] Worthington E. L. Jr. Witvliet C. V. Pietrini P. Miller A. J. (2007b). Forgiveness, health, and well-being: a review of evidence for emotional versus decisional forgiveness, dispositional forgivingness, and reduced unforgiveness. *J. Behav. Med.* 30 291–302. 10.1007/s10865-007-9105-8 17453329

[B40] Wu Y. Wen Z. (2011). Item parceling strategies in structural equation modeling [in Chinese]. *Adv. Psychol. Sci.* 19 1859–1867. 10.3724/SP.J.1042.2011.01859

[B41] Yang Y. Wang D.-F. (2007). Retest of the bidimensional model of the Rosenberg self-esteem scale [in Chinese]. *Chin. Ment. Health J.* 21 603–605. 10.3321/j.issn:1000-6729.2007.09.007

[B42] Yao S. Chen J. Yu X. Sang J. (2016). Mediator roles of interpersonal forgiveness and self-forgiveness between self-esteem and subjective well-being. *Curr. Psychol.* 36 585–592. 10.1007/s12144-016-9447-x

[B43] Zeigler-Hill V. Clark C. B. Beckman T. E. (2011). Fragile self-esteem and the interpersonal circumplex: are feelings of self-worth associated with interpersonal style? *Self Identity* 10 509–536. 10.1080/15298868.2010.497376

[B44] Zhang H.-X. Gu C.-H. (2009). The relationship between forgiveness and personal characteristics and environmental events. *Adv. Psychol. Sci.* 17 774–779.

[B45] Zhang X.-Y. Gao D.-G. Fu H. (2011). Dialectical thinking reduces aggressive tendencies [in Chinese]. *Acta Psychol. Sin.* 43 42–51. 10.3724/SP.J.1041.2011.00042

